# Comprehensive Proteomics Profiling Identifies Patients With Late Gadolinium Enhancement on Cardiac Magnetic Resonance Imaging in the Hypertrophic Cardiomyopathy Population

**DOI:** 10.3389/fcvm.2022.839409

**Published:** 2022-06-17

**Authors:** Bradley S. Lander, Yanling Zhao, Kohei Hasegawa, Mathew S. Maurer, Albree Tower-Rader, Michael A. Fifer, Muredach P. Reilly, Yuichi J. Shimada

**Affiliations:** ^1^Division of Cardiology, Department of Medicine, Columbia University Irving Medical Center, New York, NY, United States; ^2^Department of Surgery, Columbia University Irving Medical Center, New York, NY, United States; ^3^Department of Emergency Medicine, Massachusetts General Hospital, Harvard Medical School, Boston, MA, United States; ^4^Cardiology Division, Department of Medicine, Massachusetts General Hospital, Harvard Medical School, Boston, MA, United States; ^5^Irving Institute for Clinical and Translational Research, Columbia University Irving Medical Center, New York, NY, United States

**Keywords:** hypertrophic cardiomyopathy, late gadolinium enhanced (LGE), myocardial fibrosis, proteomics, cardiac magnetic resonance (MRI)

## Abstract

**Introduction:**

In hypertrophic cardiomyopathy (HCM), late gadolinium enhancement (LGE) on cardiac magnetic resonance imaging (CMR) represents myocardial fibrosis and is associated with sudden cardiac death. However, CMR requires particular expertise and is expensive and time-consuming. Therefore, it is important to specify patients with a high pre-test probability of having LGE as the utility of CMR is higher in such cases. The objective was to determine whether plasma proteomics profiling can distinguish patients with and without LGE on CMR in the HCM population.

**Materials and Methods:**

We performed a multicenter case-control (LGE vs. no LGE) study of 147 patients with HCM. We performed plasma proteomics profiling of 4,979 proteins. Using the 17 most discriminant proteins, we performed logistic regression analysis with elastic net regularization to develop a discrimination model with data from one institution (the training set; *n* = 111) and tested the discriminative ability in independent samples from the other institution (the test set; *n* = 36). We calculated the area under the receiver-operating-characteristic curve (AUC), sensitivity, and specificity.

**Results:**

Overall, 82 of the 147 patients (56%) had LGE on CMR. The AUC of the 17-protein model was 0.83 (95% confidence interval [CI], 0.75–0.90) in the training set and 0.71 in the independent test set for validation (95% CI, 0.54–0.88). The sensitivity of the training model was 0.72 (95% CI, 0.61–0.83) and the specificity was 0.78 (95% CI, 0.66–0.90). The sensitivity was 0.71 (95% CI, 0.49–0.92) and the specificity was 0.74 (95% CI, 0.54–0.93) in the test set. Based on the discrimination model derived from the training set, patients in the test set who had high probability of having LGE had a significantly higher odds of having LGE compared to those who had low probability (odds ratio 29.6; 95% CI, 1.6–948.5; *p* = 0.03).

**Conclusions:**

In this multi-center case-control study of patients with HCM, comprehensive proteomics profiling of 4,979 proteins demonstrated a high discriminative ability to distinguish patients with and without LGE. By identifying patients with a high pretest probability of having LGE, the present study serves as the first step to establishing a panel of circulating protein biomarkers to better inform clinical decisions regarding CMR utilization.

## Introduction

Hypertrophic cardiomyopathy (HCM) is among the most common inherited cardiac diseases (1). The combined prevalence of clinically expressed HCM and genetic carrier status is approximately 1 in 200 individuals in the United States (1). Patients with HCM are at risk of sudden cardiac death (SCD), yet identifying the patients at highest risk of this feared outcome remains a challenge (2–4).

Late gadolinium enhancement (LGE) on cardiac magnetic resonance imaging (CMR) represents myocardial fibrosis (5–7). In patients with HCM, LGE has been associated with an increased risk of SCD from ventricular arrhythmias (8–11). The appropriate use of implantable cardioverter-defibrillators (ICD) has reduced disease-specific mortality (12–15). Therefore, identifying LGE on CMR is critical to reducing HCM-specific mortality by facilitating ICD implantation in high-risk patients. Although CMR allows clinicians to identify LGE and patients who are at higher risk of developing SCD, it is not widely accessible, requires particular expertise for interpretation and is relatively expensive and time-consuming compared to other imaging modalities. Moreover, careful assessment of the risk-benefit balance of CMR is required in patients with claustrophobia or chronic kidney disease and pediatric patients who may require sedation or intubation during CMR (16). As a result, it is clinically indispensable to accurately determine which patients with HCM would have high pre-test probability of having LGE as the utility of CMR is higher in such cases.

Proteomics profiling is a recently developed technology that simultaneously measures the concentrations of thousands of proteins with as little as 65 microliters of blood and has been successfully used to discover biomarkers with high discriminative value in identifying HCM (17). Small studies have suggested that certain biomarkers may be associated with LGE on CMR (18–29). However, a comprehensive analysis with a high-throughput proteomics platform has not yet been performed. Thus, the purpose of this study was to test the hypothesis that plasma proteomics profiling can distinguish patients with and without LGE on CMR and identify signaling pathways associated with LGE in the HCM population. Development of a small panel of circulating protein biomarkers associated with LGE in HCM would help clinicians more precisely identify which patients with HCM should undergo CMR.

## Materials and Methods

### Study Design and Sample

We designed a case-control study in the HCM population between cases with LGE and controls without LGE. These patients were enrolled from the HCM programs at Columbia University Irving Medical Center (CUIMC) (New York, NY) and Massachusetts General Hospital (MGH) (Boston, MA) between October 13, 2015 and December 11, 2018 and were consecutively included in this study if a cardiac MRI and plasma proteomics profiling were performed. The diagnosis of HCM was established by echocardiographic evidence of left ventricular (LV) hypertrophy (maximum LV wall thickness ≥ 15 mm) that was out of proportion to the degree of systemic loading conditions and not explained by other diseases capable of producing similar findings (i.e., HCM phenocopies such as Fabry disease or cardiac amyloidosis). For patients with a family history of HCM, LV wall thickness ≥ 13 mm was considered diagnostic of HCM (30). Genetic variants classified as “definitely pathogenic” or “likely pathogenic” were considered a positive genotype whereas “variant of uncertain significance,” “likely benign” and “benign” were considered as negative genotype. We excluded patients with conditions that could lead to LV remodeling that may mimic HCM, such as aortic stenosis, subaortic membrane and exercise induced cardiac remodeling. The training set to derive the discrimination model consisted of patients from MGH. The independent test set for validation was based on patient data from CUIMC. The Mass General Brigham Institutional Review Board and that of CUIMC approved the study protocol and all participants provided written informed consent.

### Blood Sample Processing and Proteomics Profiling

Venous blood specimens were drawn at the time of an outpatient clinic visit. Samples were collected in K_2_EDTA-treated tubes and centrifuged for 10 min at 3,100 rpm. The supernatant plasma was aliquot and immediately frozen at −80 degree Celsius (17).

Proteomics profiling was performed using the SomaScan assay (SOMALogic, Inc., Boulder, CO) (17, 31, 32). This is a tool for proteomics profiling that is highly multiplexed, sensitive, quantitative and reproducible. The assay measures plasma protein concentrations, from femtomolar to micromolar, with an excellent level of reproducibility – the median coefficient of variation is 4.6% (17, 31, 32). The assay's performance is similar to that of sandwich enzyme linked immunosorbent assay and is especially useful to accurately measure concentrations of low-abundance proteins that conventional liquid chromatography/mass spectrometry cannot detect (17, 31–33). Other details of the SomaScan assay have been previously published (17, 31, 32).

### Cardiac Imaging

Two-dimensional and Doppler echocardiographic studies were performed with iE33 (Philips Medical Systems, Andover, Massachusetts) to obtain the clinical parameters presented in Table 1 using standard definitions (34, 35). Peak LV outflow tract gradient was measured with continuous-wave Doppler.

**Table 1:** Baseline clinical characteristics of the study sample.

Characteristics^*^	**LGE (+)**	**LGE (-)**	***P*** **value**
	**(*n =* 82)**	**(*n =* 65)**	
**Demographics**			
Age (year)	56 ± 15	58 ± 16	0.57
Male	60 (73)	39 (60)	0.13
Body mass index (kg/m^2^)	30 ± 6	31 ± 9	0.31
NYHA functional class ≥2	29 (35)	25 (39)	0.83
**Race**			0.67
European ancestry	70 (85)	54 (83)	
African American	1 (1)	3 (5)	
Asian	2 (2)	1 (2)	
Other or unidentified	9 (11)	7 (11)	
**Medical history**			
Prior AF	23 (28)	13 (20)	0.35
Prior VT/VF	3 (4)	2 (3)	>0.99
Prior non-sustained VT	21 (26)	7 (11)	0.03
Prior syncope	10 (12)	11 (17)	0.56
Family history of sudden cardiac death	9 (11)	5 (8)	0.58
Family history of HCM	20 (24)	16 (25)	>0.99
Obstructive HCM	39 (48)	33 (51)	0.83
Prior septal myectomy	12 (15)	15 (23)	0.27
Prior alcohol septal ablation	5 (6)	2 (3)	0.46
**Medications**			
β-blocker	56 (68)	39 (60)	0.38
Calcium channel blocker	18 (22)	15 (23)	>0.99
ACE inhibitor	9 (11)	5 (8)	0.58
ARB	12 (15)	14 (22)	0.38
**Diuretic**			
Loop diuretic	6 (7)	2 (3)	0.30
Thiazide	6 (7)	5 (8)	>0.99
Potassium sparing diuretic	4 (5)	2 (3)	0.69
Disopyramide	3 (4)	4 (6)	0.70
Amiodarone	4 (5)	2 (3)	0.69
**Blood pressure**			
Systolic blood pressure (mmHg)	124 ± 17	128 ± 17	0.24
Diastolic blood pressure (mmHg)	74 ± 11	74 ± 11	0.96
**Echocardiographic measurements**			
Left atrial diameter (mm)	35 ± 15	32 ± 17	0.29
Interventricular septum thickness (mm)	13 ± 8	11 ± 6	0.03
Posterior wall thickness (mm)	9 ± 5	9 ± 5	0.37
Maximal LV wall thickness (mm)	22 ± 6	18 ± 4	<0.001
LV outflow tract gradient (mmHg) at rest	0 [0–22]	12 [0–33]	0.24
LV outflow tract gradient (mmHg) with Valsalva maneuver	25 [1–73]	26 [0–72]	0.89
LV ejection fraction (%)	68 ± 11	68 ± 8	0.95
LV end-diastolic diameter (mm)	36 ± 17	31 ± 18	0.12
LV end-systolic diameter (mm)	25 ± 7	24 ± 9	0.54
Systolic anterior motion of mitral valve leaflet	26 (32)	25 (39)	0.50
Degree of mitral regurgitation^†^	1.5 [1–2]	1.5 [1–2]	0.41
**Genetic testing** (*n =* 84)			
Pathogenic or likely pathogenic	19 (23)	6 (9)	0.03

* *Data are expressed as number (percentage), mean ± standard deviation, or median [interquartile range]*.

† *Degree of mitral regurgitation was converted to numerical values according to the following rule: none = 0, trace = 1, trace to mild = 1.5, mild = 2, mild to moderate = 2.5, moderate = 3, moderate to severe = 3.5, severe = 4. ACE, angiotensin-converting enzyme; AF, atrial fibrillation; ARB, angiotensin II receptor blocker; HCM, hypertrophic cardiomyopathy; LGE, late gadolinium enhancement; LV, left ventricular; NYHA, New York Heart Association; VT/VF, ventricular tachycardia or ventricular fibrillation*.

CMR was ordered at the discretion of the treating physicians. CMR studies were performed on a 1.5-T field strength scanner (HDXt platform, General Electric Healthcare, Milwaukee, Wisconsin) with a dedicated 8-channel cardiac-coil. The imaging protocol included localizer images with cine-balanced steady-state free precession imaging in the short axis, paraseptal long axis, horizontal long axis and 3-chamber views. The myocardial late enhancement sequences were performed in LV short axis and radial long axis 8 to 15 min after the 0.2 mmol/kg injection of intravenous gadopentetate demglumine (Magnevist, Bater HealthCare Pharmaceuticals Inc., Wayne, New Jersey). Short axis late enhancement views were obtained with both 2-dimensional single slice per breath-hold imaging and 3-dimensional volumetric ventricular imaging. Inversion times were determined on an individual basis to null the normal myocardial signal.

The images were reviewed by expert readers using dedicated CMR analysis software (cmr^42^, Circle Cardiovascular Imaging Inc., Calgary, Alberta, Canada). Late myocardial enhancement images were analyzed using 2-dimensional views and coregistered 3-dimensional and long axis views for correlation when indicated (36). The presence and absence of LGE was determined by the reading cardiac radiologist. CMR readers were blinded to the results of plasma proteomics.

### Univariable Analysis

We presented continuous variables as mean ± standard deviation if normally distributed and as median [interquartile range] if not normally distributed. To compare clinical characteristics between patients with LGE and without LGE, we used the unpaired Student's *t-*test for normally distributed continuous variables, the Mann-Whitney-Wilcoxon test for other continuous and ordinal variables (e.g., degree of mitral regurgitation) and the χ^2^ test for categorical variables.

### Development of a 17-Protein Model to Distinguish Patients With and Without LGE

Logistic regression with elastic net regularization was used with a plan to identify a set of 15 candidate proteins with the greatest potential to discriminate patients with and without LGE (e.g., the 15 most discriminant proteins). This method was chosen due to its advantage in addressing issues when the number of predictors (4,979) is much larger than the number of observations (147) (37, 38). The logistic regression with elastic net regularization was trained using a 5-fold cross-validation methods in the training set (i.e., patients followed at MGH, 111 of 147 patients). To preprocess the variables for the elastic net model, we performed sample-wise normalization using the median of all protein concentrations in each sample followed by protein-wise log transformation and Pareto scaling in each protein concentration. We then created a hyperparameter tuning grid to identify best hidden component and threshold parameter using the R *caret* and *glmnet* packages. Ultimately, 3 proteins were tied for the 15^th^ most discriminative protein, thus leading to the inclusion of a total of 17 proteins in the model. We tested the discriminative ability of the model in the independent test set (i.e., patients followed at CUIMC, 36 of 147 patients) to test the performance of the model developed from the training set. We calculated the area under the receiver-operating-characteristic curve (AUC), sensitivity, specificity, positive predictive value and negative predictive value as indicators of the model's discriminative ability.

### Pathway Analysis

We performed pathway analysis to identify canonical pathways that are dysregulated (i.e., either upregulated or downregulated) between patients with and without LGE. We used the 144 most discriminant proteins based on a *p*-value of < 0.05 with univariable analysis. We subsequently determined the associations among the 144 most discriminant proteins and canonical pathways in the Kyoto Encyclopedia of Genes and Genomes (KEGG) (39). Significance was based on the ratio of the number of proteins within the 144 most discriminant proteins that map to a canonical pathway divided by the number of proteins that belong to the pathway (39). We considered a pathway as positive (i.e., dysregulated) if the false discovery rate (FDR) was <0.05 and there were at least 3 associated proteins (40). We used STRING version 11.5 (String Consortium, Europe) for the pathway analysis and conducted other analyses with R version 4.1.0 (R Foundation for Statistical Computing, Vienna, Austria) (39).

## Results

In total, the analysis included 147 patients with HCM (82 with LGE and 65 without LGE). The training set used to develop the discrimination model was comprised of 111 patients from MGH, of which 65 patients had LGE and 46 patients did not have LGE. The independent test set used for validation was comprised of 36 patients from CUIMC, of which 17 patients had LGE and 19 patients did not have LGE.

Baseline characteristics are presented in Table 1. Patients with LGE had a greater degree of both maximal LV wall and interventricular septal wall thickness, were more likely to have had prior non-sustained ventricular tachycardia (NSVT) and a pathogenic or likely pathogenic genetic variant when compared to patients without LGE. Other demographic and clinical characteristics were similar between the 2 groups.

**Figure 1:**
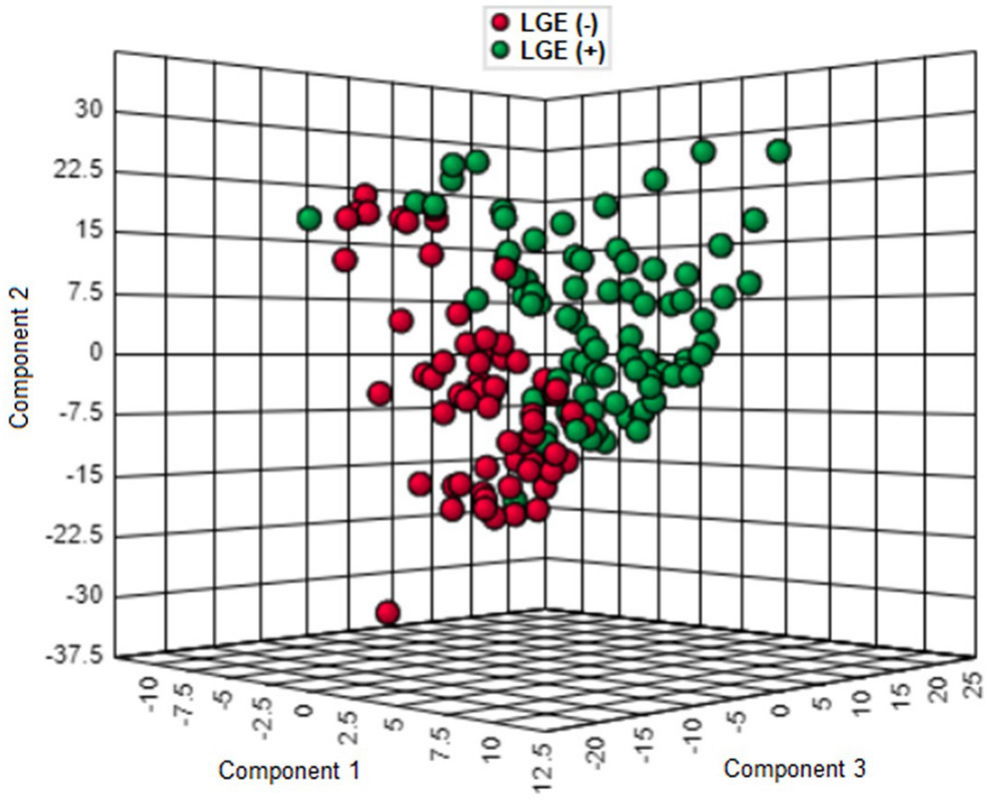
Three-dimensional score plot of proteomics profiling in patients with and without late gadolinium enhancement in the hypertrophic cardiomyopathy population. Each green circle represents the proteomic profile of a patient with LGE. Each red circle corresponds to that of a patient without LGE. HCM, hypertrophic cardiomyopathy; LGE, late gadolinium enhancement.

**Figure 2:**
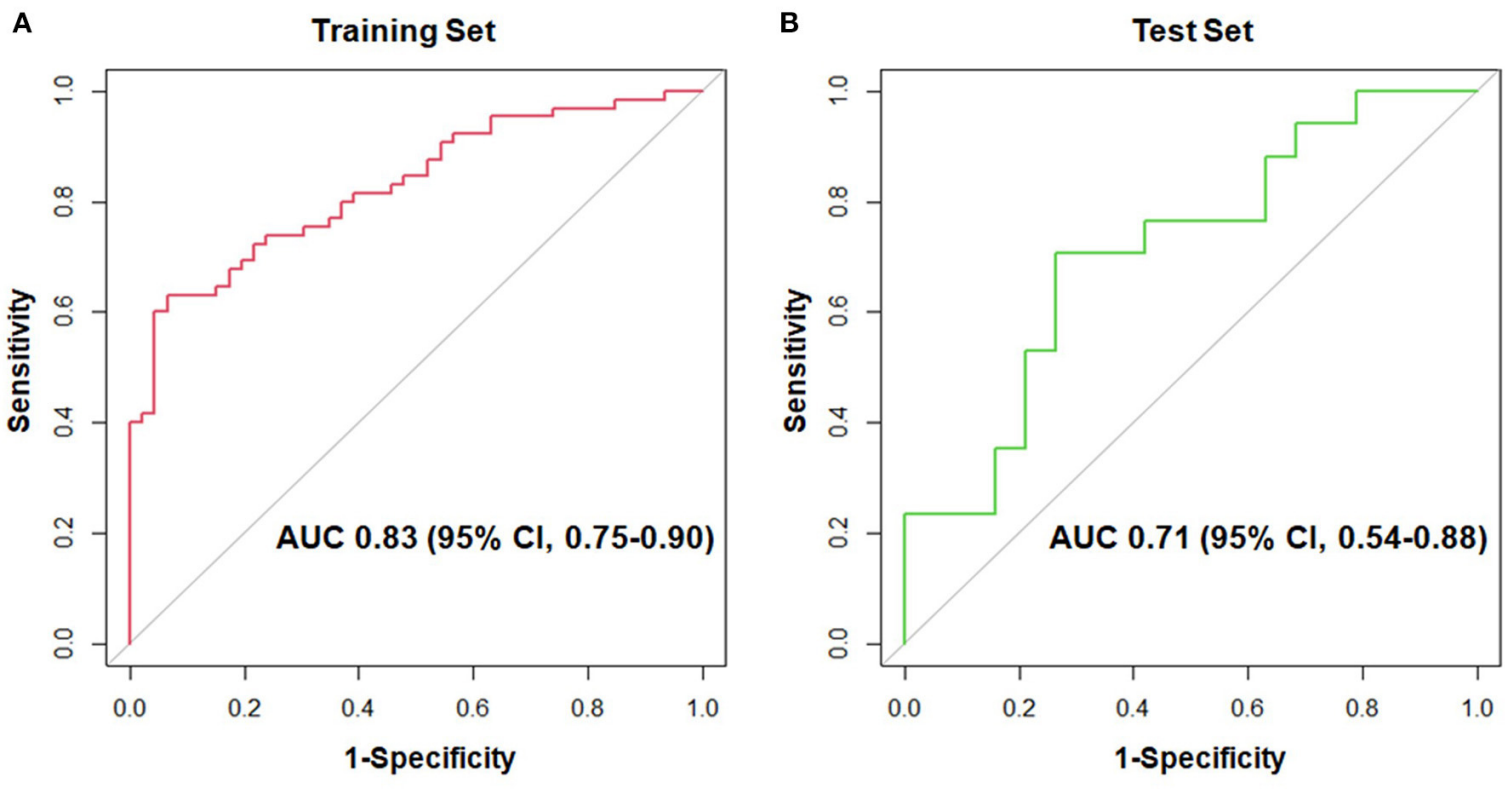
Receiver-operating-characteristic curves for the 17-protein model to distinguish patients with and without late gadolinium enhancement in the hypertrophic cardiomyopathy population. **(A)** shows the receiver-operating-characteristic curve in the training set, whereas **(B)** displays that in the test set. AUC, area under the receiver-operating-characteristic curve; CI, confidence interval.

**Figure 3:**
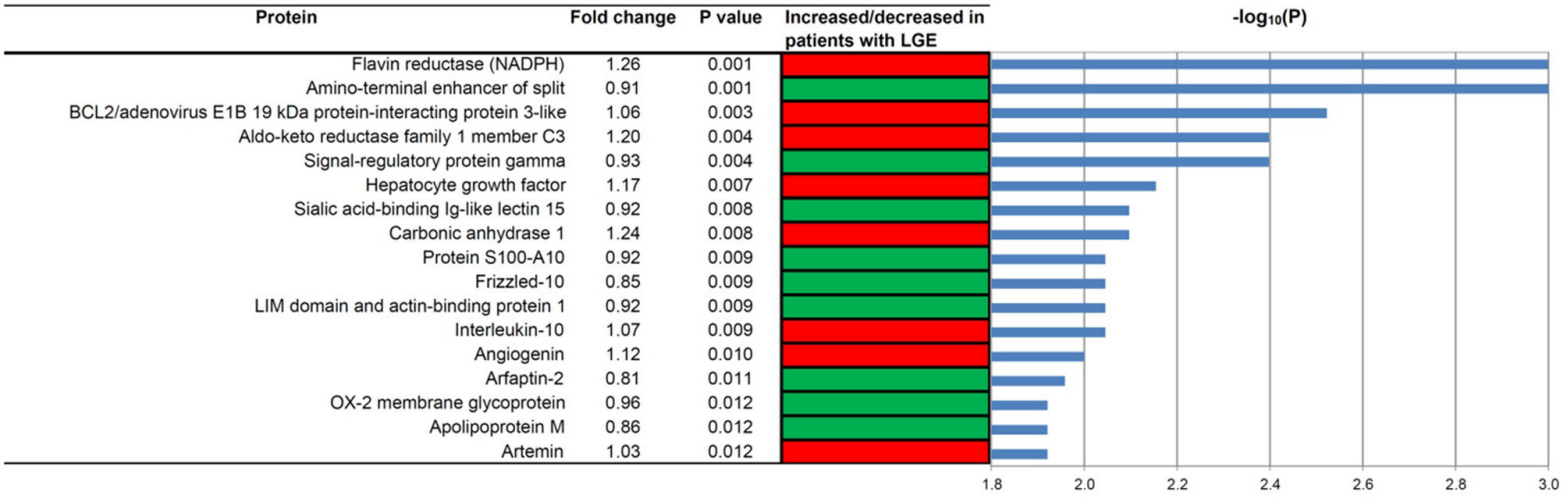
The 17 most discriminant proteins to distinguish patients with and without late gadolinium enhancement in the hypertrophic cardiomyopathy population. A red box indicates that the protein concentration was increased in patients with LGE, while a green box means that the concentration was decreased in patients with LGE. *P* values were computed using the Mann-Whitney-Wilcoxon test. Fold change was calculated by dividing the median in the cases by the median in the controls. HCM, hypertrophic cardiomyopathy; LGE, late gadolinium enhancement.

As shown in Figure 1, the proteomic profiles differed between HCM patients with and without LGE. The discrimination model to distinguish LGE positivity showed high discriminative ability in the training set (AUC 0.83, 95% confidence interval [CI] 0.75–0.90; *p* < 0.001 compared to the null hypothesis of AUC = 0.5; Figure 2). The sensitivity in the training set was 0.72 (95% CI, 0.61–0.83) and the specificity was 0.78 (95% CI, 0.66–0.90). Furthermore, the discrimination model derived from the training set maintained accuracy when applied to the independent test set for validation (AUC 0.71, 95% CI 0.54–0.88, *p* = 0.03; Figure 2). The sensitivity was 0.71 (95% CI, 0.49–0.92) and the specificity was 0.74 (95% CI, 0.54–0.93; Table 2) in the test set. Based on the discrimination model derived from the training set, patients in the test set who had high probability of having LGE had significantly higher odds of having LGE compared to those who had low probability (odds ratio 29.6, 95% CI 1.6–948.5; *p* = 0.03). Figure 3 displays the 17 most discriminant proteins that were included in the discrimination model.

**Table 2:** Area under the receiver-operating-characteristic curve, sensitivity, specificity, positive predictive value and negative predictive value of the 17-protein model to distinguish patients with and without late gadolinium enhancement.

**Cohort**	**AUC** **(95% CI)**	**Sensitivity** **(%)** **(95% CI)**	**Specificity (%)** **(95% CI)**	**PPV (%)** **(95% CI)**	**NPV (%) (95% CI)**
**Training set**	0.83 (0.75–0.90)	72 (61–83)	78 (66–90)	82 (73–92)	67 (54–79)
**Test set**	0.71 (0.54–0.88)	71 (49–92)	74 (54–93)	71 (49–92)	74 (54–94)

*AUC, area under the receiver-operating-characteristic curve; CI, confidence interval; NPV, negative predictive value; PPV, positive predictive value*.

Pathway analysis using the 144 most discriminant proteins demonstrated dysregulation in 15 pathways with FDR < 0.05 (Table 3). These included pathways that have been recognized to be dysregulated in HCM, such as those involved in inflammation (interleukin-17, cytokine-cytokine receptor interaction) as well as sugar and amino acid metabolism. Moreover, the list of dysregulated pathways contained pathways that were previously unrecognized to be dysregulated in HCM with LGE – e.g., the RIG-I-like receptor signaling pathway and the PI3K-Akt signaling pathway.

**Table 3:** Pathways that are differentially regulated between patients with and without late gadolinium enhancement in the hypertrophic cardiomyopathy population.

**Pathway description**	**Number of matching proteins**	**Number of proteins in the pathway**	**False discovery rate**
Glycolysis/gluconeogenesis	8	68	0.00001
Metabolic pathways	27	1,250	0.00003
Carbon metabolism	9	116	0.00003
Biosynthesis of amino acids	7	72	0.0001
Pentose phosphate pathway	5	30	0.0002
Fructose and mannose metabolism	5	33	0.0002
Phenylalanine metabolism	3	17	0.01
IL-17 signaling pathway	5	92	0.01
RIG-I-like receptor signaling pathway	4	70	0.03
Tyrosine metabolism	3	36	0.03
Glycine, serine and threonine metabolism	3	39	0.03
Cytokine-cytokine receptor interaction	7	263	0.03
Hypertrophic cardiomyopathy	4	81	0.03
PI3K-Akt signaling pathway	8	348	0.04
Dilated cardiomyopathy	4	88	0.04

*IL, interleukin; PI3K, phosphoinositide 3-kinase; RIG-I, retinoic acid-inducible gene-I*.

## Discussion

### Summary of Findings

In the present multi-center case-control study of 82 cases with LGE and 65 controls without LGE on CMR in the HCM population, comprehensive proteomics profiling of 4,979 proteins demonstrated a good discriminative ability to distinguish patients with and without LGE. Furthermore, pathway analysis displayed previously recognized (e.g., inflammation, sugar and amino acid metabolism) and newly recognized (e.g., RIG-I-like receptor signaling, PI3K-Akt signaling) pathways that were dysregulated in patients with LGE.

### Results in Context

LGE in HCM represents myocardial fibrosis (5–7) and has been associated with an increased risk of SCD from ventricular arrhythmias, which can be effectively aborted by ICD (8–11). By subsequently facilitating ICD implantation, identifying high-risk features such as LGE on CMR contributes to reduced disease-specific mortality (12–15). However, in certain circumstances, patients are unable to easily undergo CMR with gadolinium enhancement for risk stratification due to accessibility and expertise required to conduct and interpret the test and patient-specific factors such as claustrophobia and chronic kidney disease (41–43). Therefore, it is important to identify patients with HCM who have a high pretest probability of LGE, because pursuing CMR, despite potential barriers, would be more likely to change clinical management in this HCM subpopulation.

The prevalence of LGE in the present study is consistent with prior studies that suggest a pooled prevalence of LGE of approximately 60% (44). Prior studies have used various methods to predict LGE on CMR. A prior study reported that a clinical model including a history of NSVT, reduced LV systolic function and maximal echocardiographic LV wall thickness had a high discriminative ability to predict extensive LGE. Nevertheless, the study's exclusion of patients at high risk for SCD limits the generalizability of the study (29). Thus far, 2 studies attempted to estimate the likelihood and extent of LGE based on electrocardiographic findings (45, 46). However, 1 study was limited by a small sample size (42 patients including controls) and young age (7–31 years), making the inferences less applicable to older patients seen in adult cardiology practices (45). The other study used the Selvester QRS score and showed a high degree of accuracy to determine the presence and extent of LGE but was limited by an extensive scoring system and the need for automated software (46).

In addition to these clinical and electrocardiogram-based prediction models, the association between plasma circulating biomarkers such as cardiac troponin, natriuretic peptides and markers of collagen turnover have been studied in the context of LGE in HCM (21, 23, 24, 47). Higher concentrations of cardiac troponin have been associated with LGE (21, 22, 24, 25, 29, 47). Other studies have shown elevated concentrations of midregional pro-adrenomedullin (27) and matrix metalloproteinase 9 (18) and lower levels of apelin to be associated with LGE (23). Concentrations of serum N-terminal pro-B-type natriuretic peptide and B-type natriuretic peptide have been associated with LGE in some studies but not in others on multivariable analysis (24, 47). Taken together, these prior studies collectively support the importance of identifying protein biomarkers of LGE in HCM that are easily obtained in a non-invasive manner (e.g., blood). In this context, the present study serves as the first to apply comprehensive proteomics approach to specify novel circulating biomarkers of LGE in HCM.

### Application of Proteomics Profiling to Biomarker Discovery in Cardiovascular Diseases

Proteomics profiling using the SomaScan assay has previously been utilized to identify novel plasma circulating biomarkers associated with cardiovascular diseases and cardiometabolic risk (e.g., the Framingham Heart Study) (48). Furthermore, proteomics profiling has been applied to several cardiovascular conditions including coronary artery disease (48–50), hypertension (51), heart failure and cardiomyopathies (52–56). Our group and others have previously demonstrated the role of plasma proteomics profiling in distinguishing HCM from healthy controls (57) and other cardiovascular conditions (17, 58). A recent small study has also shown differences in proteomics profiling among patients with HCM before and after surgical myectomy (59). On the whole, these studies support the role of proteomics profiling to identify novel biomarkers in a variety of cardiovascular conditions. Our current study of comprehensive proteomics profiling adds to the literature by demonstrating that the discrimination model using a small number (17 proteins) of plasma biomarkers has good accuracy to detect LGE in the HCM population. The potential clinical utility of such a small panel of plasma circulating biomarkers is further underscored by the availability of rapid and low-cost methods to determine plasma protein concentrations (e.g., sandwich enzyme linked immunosorbent assay).

### Signaling Pathways Associated With LGE on CMR in HCM

Prior studies have demonstrated several dysregulated signaling pathways associated with cardiac hypertrophy, including those related to glycolysis (60–62), the pentose phosphate pathway (60, 63), fructose and mannose metabolism (61, 62, 64, 65), tyrosine metabolism (57) and glycine, serine and threonine metabolism (66). These metabolic pathways were also found to be dysregulated in the present study, suggesting that these pathways contribute not only to the development of LV hypertrophy but also to the progression to LV fibrosis in HCM.

The present study also revealed dysregulation of previously unrecognized pathways – e.g., the RIG-I-like receptor signaling, the PI3K-Akt signaling pathway (67–70) – in patients with LGE in the HCM population. The association between the PI3K-Akt pathway and LGE in HCM is an interesting finding because this pathway is upstream to the Ras-MAPK pathway, upregulation of which has been shown to cause HCM-like cardiac changes in RASopathies such as Noonan syndrome (71–75). Recent proteomics studies have suggested that the Ras-MAPK pathway is upregulated in patients with HCM and is associated with larger left atrial diameters and more severe New York Heart Association functional classes (17, 58). While the PI3K-Akt pathway has been previously associated with physiologic cardiac hypertrophy (67–69), the newly observed association with LGE in HCM has particularly relevant clinical implications because the downstream Ras-MAPK pathway is modifiable. Specifically, the HCM-like cardiac phenotype in RASopathies can be mitigated by Ras-MAPK inhibition (75–77). Moreover, this pathway has been a drug target in cancer treatment development and such data may inform future applicability to cardiovascular disease (78). Taken together with prior reports, the observation in the present study indicates that the PI3K-Akt pathway and its downstream Ras-MAPK pathway may play a role not only in HCM pathogenesis but also in progression to LV fibrosis. Our findings also suggest that targeting the upstream P13K-Akt pathway may be another worthwhile focus for future drug development as it relates to LV fibrosis in HCM and the availability of inhibitors specific to the pathway further underscores the potential utility of such efforts (78, 79).

### Strengths of the Present Study

We took multiple measures to minimize false positive and negative findings and to enhance the internal and external validity of the study. First, we derived a proteomics-based discrimination model from the training set of patients followed at MGH and validated its discriminative ability in an independent test set of patients followed at CUIMC. The observation that the proteomics-based model to predict LGE maintained good accuracy in the independent test set underscores the robustness of the model and the external validity of the inferences from the present study. Second, to reduce false positive declarations, we used an FDR threshold of 0.05 to determine the significance of pathway dysregulation. Using FDR restricts the study-wide rate of false positives. An FDR threshold of 0.05 ensures that <1 of 20 pathways that are declared positive are false positives. Moreover, by using pathway analysis, we strengthen the biological plausibility and reduce the risk of false positive discovery given that the proteins are interconnected versus isolated findings using a univariable analysis (40). Third, with respect to false negative findings, our list of differentially regulated proteins and pathways included those known to be dysregulated in cardiac hypertrophy (e.g., the KEGG pathway named “hypertrophic cardiomyopathy”) and other pathways known to be involved in HCM pathogenesis (e.g., inflammation, sugar and amino acid metabolism). These pathways serve as “positive controls” in our study and further support the robustness of plasma proteomics to identify signaling pathways that are differentially regulated between patients with and without LGE on CMR in the HCM population. Finally, our study utilized the most comprehensive (~5000 proteins) proteomics profiling to date (17, 80), thus reducing the risk of false negatives (ie: failure to identify important protein biomarkers and pathways).

### Potential Limitations

There are several potential limitations to the current study. First, LGE was a binary variable and quantification was not performed to identify the extent of LGE. Second, no association with subsequent clinical outcomes (e.g., SCD) was evaluated. Third, the study sample consisted of patients who were enrolled at tertiary care centers and underwent CMR. Therefore, the inferences may not be generalizable to populations with less severe clinical manifestations or those who did not undergo CMR. However, limiting enrollment to 2 centers enabled strict control and standardization of the protocol which are indispensable components of accurate proteomics profiling and CMR. Fourth, temporality or causality between differentially regulated pathways and LGE in HCM was not assessed. Fifth, not all patients with HCM underwent genetic testing. Sixth, myocardial samples were not available and as such, direct analysis with tissue specimens could not be performed. Seventh, the number of patients included in the test set was relatively small and the negative predictive value was modest, and therefore negative prediction by proteomics profiling did not completely rule out LGE on CMR. Nevertheless, the current analysis serves as a proof-of-concept study for future investigations to further improve the predictive ability. And finally, although the sample size was larger than most prior studies and we used the aforementioned methods to reduce the chance of false positive discovery, the possibility of false positive discovery remains.

## Conclusions

The present study demonstrated, for the first time, the role of comprehensive proteomics profiling to distinguish patients with and without LGE on CMR in the HCM population and revealed signaling pathways associated with LGE. By identifying patients with a high pretest probability of having LGE, the present study would serve as the first step to establishing a panel of circulating protein biomarkers to better inform clinical decisions between patients and physicians regarding CMR utilization when the risk-benefit calculation of CMR is balanced. Our work also exhibited that multiple pathways, both known and novel, were upregulated in patients with LGE. These findings should facilitate further investigations into the underlying molecular mechanisms through which genetic mutations lead to the development of LGE in patients with HCM and pathways that may be targeted by future pharmacotherapies.

## Data Availability Statement

The data that support the findings of this study are available from the corresponding author upon reasonable request.

## Ethics Statement

The studies involving human participants were reviewed and approved by the Columbia University Irving Medical Center Institutional Review Board and the Mass General Brigham Institutional Review Board. The patients/participants provided their written informed consent to participate in this study.

## Author Contributions

BL, YZ, KH, and YS contributed to conception and design, analysis, and interpretation of the data, as well as manuscript preparation and revision. MM, AT-R, MF, and MR contributed to interpretation of the data and manuscript revision. All authors have read and approved the final manuscript. All authors contributed to the article and approved the submitted version.

## Funding

YS was supported by research grants from the National Institute of Health (Bethesda, MD; R01 HL157216), American Heart Association (Dallas, TX) National Clinical and Population Research Awards and Career Development Award, The Feldstein Medical Foundation (Clifton, NJ) Medical Research Grant, Korea Institute of Oriental Medicine (Daejeon, Republic of Korea), and Columbia University (New York, NY) Irving Medical Center Irving Institute for Clinical and Translational Research Precision Medicine Pilot Award as well as Columbia University Irving Medical Center Lewis Katz Cardiovascular Research Award.

## Conflict of Interest

The authors declare that the research was conducted in the absence of any commercial or financial relationships that could be construed as a potential conflict of interest.

## Publisher's Note

All claims expressed in this article are solely those of the authors and do not necessarily represent those of their affiliated organizations, or those of the publisher, the editors and the reviewers. Any product that may be evaluated in this article, or claim that may be made by its manufacturer, is not guaranteed or endorsed by the publisher.

## References

[1] Semsarian C Ingles J Maron MS Maron BJ. New perspectives on the prevalence of hypertrophic cardiomyopathy. J Am Coll Cardiol. (2015) 65:1249–54. 10.1016/j.jacc.2015.01.019 25814232

[2] Ommen SR Mital S Burke MA Day SM Deswal A Elliott P. 2020 AHA/ACC guideline for the diagnosis and treatment of patients with hypertrophic cardiomyopathy: A report of the American College of Cardiology/American Heart Association Joint Committee on Clinical Practice Guidelines. J Am Coll Cardiol. (2020) 76:e159–240. 10.1016/j.jacc.2020.08.045 33229116

[3] Elliott PM Anastasakis A Borger MA Borggrefe M Cecchi F Charron P. 2014 ESC guidelines on diagnosis and management of hypertrophic cardiomyopathy: the task force for the diagnosis and management of hypertrophic cardiomyopathy of the European Society of Cardiology (ESC). Eur Heart J. (2014) 35:2733–79. 10.1093/eurheartj/ehu284 25173338

[4] Maron MS Rowin EJ Wessler BS Mooney PJ Fatima A Patel P. Enhanced American College of Cardiology/American Heart Association strategy for prevention of sudden cardiac death in high-risk patients with hypertrophic cardiomyopathy. JAMA Cardiol. (2019) 4:644–57. 10.1001/jamacardio.2019.1391 31116360 PMC6537832

[5] Kuruvilla S Adenaw N Katwal AB Lipinski MJ Kramer CM Salerno M. Late gadolinium enhancement on cardiac magnetic resonance predicts adverse cardiovascular outcomes in nonischemic cardiomyopathy: a systematic review and meta-analysis. Circ Cardiovasc Imaging. (2014) 7:250–8. 10.1161/CIRCIMAGING.113.001144 24363358 PMC4007583

[6] Moon JCC Reed E Sheppard MN Elkington AG Ho SY Burke M. The histologic basis of late gadolinium enhancement cardiovascular magnetic resonance in hypertrophic cardiomyopathy. J Am Coll Cardiol. (2004) 43:2260–4. 10.1016/j.jacc.2004.03.035 15193690

[7] Kwon DH Smedira NG Rodriguez ER Tan C Setser R Thamilarasan M. Cardiac magnetic resonance detection of myocardial scarring in hypertrophic cardiomyopathy: correlation with histopathology and prevalence of ventricular tachycardia. J Am Coll Cardiol. (2009) 54:242–9. 10.1016/j.jacc.2009.04.026 19589437

[8] Suk T Edwards C Hart H Christiansen JP. Myocardial scar detected by contrast-enhanced cardiac magnetic resonance imaging is associated with ventricular tachycardia in hypertrophic cardiomyopathy patients. Heart Lung Circ. (2008) 17:370–4. 10.1016/j.hlc.2008.03.080 18562248

[9] Weng Z Yao J Chan RH He J Yang X Zhou Y. Prognostic value of LGE-CMR in HCM: A meta-analysis. JACC Cardiovasc Imaging. (2016) 9:1392–402. 10.1016/j.jcmg.2016.02.031 27450876

[10] Mentias A Raeisi-Giglou P Smedira NG Feng K Sato K Wazni O. Late gadolinium enhancement in patients with hypertrophic cardiomyopathy and preserved systolic function. J Am Coll Cardiol. (2018) 72:857–70. 10.1016/j.jacc.2018.05.060 30115224

[11] Chan RH Maron BJ Olivotto I Pencina MJ Assenza GE Haas T. Prognostic value of quantitative contrast-enhanced cardiovascular magnetic resonance for the evaluation of sudden death risk in patients with hypertrophic cardiomyopathy. Circulation. (2014) 130:484–95. 10.1161/CIRCULATIONAHA.113.007094 25092278

[12] Maron BJ Rowin EJ Casey SA Link MS Lesser JR Chan RHM. Hypertrophic Cardiomyopathy in adulthood associated with low cardiovascular mortality with contemporary management strategies. J Am Coll Cardiol. (2015) 65:1915–28. 10.1016/j.jacc.2015.02.061 25953744

[13] Maron BJ Spirito P Shen W-K Haas TS Formisano F Link MS. Implantable cardioverter-defibrillators and prevention of sudden cardiac death in hypertrophic cardiomyopathy. JAMA. (2007) 298:405–12. 10.1001/jama.298.4.405 17652294

[14] Vriesendorp PA Schinkel AFL Van Cleemput J Willems R Jordaens LJLM Theuns DAMJ. Implantable cardioverter-defibrillators in hypertrophic cardiomyopathy: patient outcomes, rate of appropriate and inappropriate interventions, and complications. Am Heart J. (2013) 166:496–502. 10.1016/j.ahj.2013.06.009 24016499

[15] Maron BJ Rowin EJ Casey SA Lesser JR Garberich RF McGriff DM. Hypertrophic cardiomyopathy in children, adolescents, and young adults associated with low cardiovascular mortality with contemporary management strategies. Circulation. (2016) 133:62–73. 10.1161/CIRCULATIONAHA.115.017633 26518766

[16] Dickstein K. Clinical utilities of cardiac MRI. e-journal Cardiol Pract [Internet]. (2008). Available online at: https://www.escardio.org/Journals/E-Journal-of-Cardiology-Practice/Volume-6/Clinical-Utilities-of-cardiac-MRI. (accessed November 7, 2021)

[17] Shimada YJ Hasegawa K Kochav SM Mohajer P Jung J Maurer MS. Application of proteomics profiling for biomarker discovery in hypertrophic cardiomyopathy. J Cardiovasc Transl Res. (2019) 12:569–79. 10.1007/s12265-019-09896-z 31278493 PMC7102897

[18] Münch J Avanesov M Bannas P Säring D Krämer E Mearini G. Serum matrix metalloproteinases as quantitative biomarkers for myocardial fibrosis and sudden cardiac death risk stratification in patients with hypertrophic cardiomyopathy. J Card Fail. (2016) 22:845–50. 10.1016/j.cardfail.2016.03.010 27018569

[19] Gawor M Spiewak M Kubik A Wróbel A Lutyńska A Marczak M. Circulating biomarkers of hypertrophy and fibrosis in patients with hypertrophic cardiomyopathy assessed by cardiac magnetic resonance. Biomarkers. (2018) 23:676–82. 10.1080/1354750X.2018.1474261 29737871

[20] Park JR Choi J-O Han HJ Chang S-A Park S-J Lee S-C. Degree and distribution of left ventricular hypertrophy as a determining factor for elevated natriuretic peptide levels in patients with hypertrophic cardiomyopathy: insights from cardiac magnetic resonance imaging. Int J Cardiovasc Imaging. (2012) 28:763–72. 10.1007/s10554-011-9876-4 21516440

[21] Hasler S Manka R Greutmann M Gämperli O Schmied C Tanner FC. Elevated high-sensitivity troponin T levels are associated with adverse cardiac remodelling and myocardial fibrosis in hypertrophic cardiomyopathy. Swiss Med Wkly. (2016) 146:w14285. 10.4414/smw.2016.14285 26999566

[22] Neubauer S Kolm P Ho CY Kwong RY Desai MY Dolman SF. Distinct subgroups in hypertrophic cardiomyopathy in the NHLBI HCM Registry. J Am Coll Cardiol. (2019) 74:2333–45. 10.1016/j.jacc.2019.08.1057 31699273 PMC6905038

[23] Zhou Y Yuan J Wang Y Qiao S. Predictive values of apelin for myocardial fibrosis in hypertrophic cardiomyopathy. Int Heart J. (2019) 60:648–55. 10.1536/ihj.18-598 31019180

[24] Kawasaki T Sakai C Harimoto K Yamano M Miki S Kamitani T. Usefulness of high-sensitivity cardiac troponin T and brain natriuretic peptide as biomarkers of myocardial fibrosis in patients with hypertrophic cardiomyopathy. Am J Cardiol. (2013) 112:867–72. 10.1016/j.amjcard.2013.04.060 23746480

[25] Moreno V Hernández-Romero D Vilchez JA García-Honrubia A Cambronero F Casas T. Serum levels of high-sensitivity troponin T: a novel marker for cardiac remodeling in hypertrophic cardiomyopathy. J Card Fail. (2010) 16:950–6. 10.1016/j.cardfail.2010.07.245 21111984

[26] Wang Y Tang Y Zou Y Wang D Zhu L Tian T. Plasma level of big endothelin-1 predicts the prognosis in patients with hypertrophic cardiomyopathy. Int J Cardiol. (2017) 243:283–9. 10.1016/j.ijcard.2017.03.162 28587741

[27] Elmas E Doesch C Fluechter S Freundt M Weiss C Lang S. Midregional pro-atrial natriuretic peptide: a novel marker of myocardial fibrosis in patients with hypertrophic cardiomyopathy. Int J Cardiovasc Imaging. (2011) 27:547–56. 10.1007/s10554-010-9704-2 20872251

[28] Roldán V Marín F Gimeno JR Ruiz-Espejo F González J Feliu E. Matrix metalloproteinases and tissue remodeling in hypertrophic cardiomyopathy. Am Heart J. (2008) 156:85–91. 10.1016/j.ahj.2008.01.035 18585501

[29] Gommans DHF Cramer GE Fouraux MA Bakker J Michels M Dieker H-J. Prediction of extensive myocardial fibrosis in nonhigh risk patients with hypertrophic cardiomyopathy. Am J Cardiol. (2018) 122:483–9. 10.1016/j.amjcard.2018.04.020 30201111

[30] McKenna WJ Spirito P Desnos M Dubourg O Komajda M. Experience from clinical genetics in hypertrophic cardiomyopathy: proposal for new diagnostic criteria in adult members of affected families. Heart. (1997) 77:130–2. 10.1136/hrt.77.2.130 9068395 PMC484661

[31] Hensley P. SOMAmers and SOMAscan – A protein biomarker discovery platform for rapid analysis of sample collections from bench top to the clinic. J Biomol Tech. (2013) 24:S5.

[32] Kraemer S Vaught JD Bock C Gold L Katilius E Keeney TR. From SOMAmer-based biomarker discovery to diagnostic and clinical applications: a SOMAmer-based, streamlined multiplex proteomic assay. PLoS ONE. (2011) 6:e26332. 10.1371/journal.pone.0026332 22022604 PMC3195687

[33] Gramolini A Lau E Liu PP. Identifying low-abundance biomarkers: aptamer-based proteomics potentially enables more sensitive detection in cardiovascular diseases. Circulation. (2016) 134:286–9. 10.1161/CIRCULATIONAHA.116.022940 27444931

[34] Lang RM Badano LP Mor-Avi V Afilalo J Armstrong A Ernande L. Recommendations for cardiac chamber quantification by echocardiography in adults: an update from the American Society of Echocardiography and the European Association of Cardiovascular Imaging. Eur Hear J Cardiovasc Imaging. (2015) 16:233–70. 10.1093/ehjci/jev014 25712077

[35] Nagueh SF Appleton CP Gillebert TC Marino PN Oh JK Smiseth OA. Recommendations for the evaluation of left ventricular diastolic function by echocardiography. J Am Soc Echocardiogr. (2009) 22:107–33. 10.1016/j.echo.2008.11.023 19187853

[36] Harrigan CJ Peters DC Gibson CM Maron BJ Manning WJ Maron MS. Hypertrophic cardiomyopathy: quantification of late gadolinium enhancement with contrast-enhanced cardiovascular MR imaging. Radiology. (2011) 258:128–33. 10.1148/radiol.10090526 21045187

[37] Kurnaz FS Hoffmann I Filzmoser P. Robust and sparse estimation methods for high-dimensional linear and logistic regression. Chemom Intell Lab Syst. (2018) 172:211–22. 10.1016/j.chemolab.2017.11.017

[38] Zhang S Zhang L Qiu K Lu Y Cai B. Variable selection in logistic regression model. Chinese J Electron. (2015) 24:813–7. 10.1049/cje.2015.10.02529610737

[39] Szklarczyk D Morris JH Cook H Kuhn M Wyder S Simonovic M. The STRING database in 2017: quality-controlled protein-protein association networks, made broadly accessible. Nucleic Acids Res. (2017) 45:D362–8. 10.1093/nar/gkw937 27924014 PMC5210637

[40] Pawitan Y Michiels S Koscielny S Gusnanto A Ploner A. False discovery rate, sensitivity and sample size for microarray studies. Bioinformatics. (2005) 21:3017–24. 10.1093/bioinformatics/bti448 15840707

[41] Thomsen HS. Gadolinium-based contrast media may be nephrotoxic even at approved doses. Eur Radiol. (2004) 14:1654–6. 10.1007/s00330-004-2379-0 15221265

[42] Ergün I Keven K Uruç I Ekmekçi Y Canbakan B Erden I. The safety of gadolinium in patients with stage 3 and 4 renal failure. Nephrol Dial Transplant. (2006) 21:697–700. 10.1093/ndt/gfi304 16326736

[43] MRI (Magnetic Resonance Imaging): Benefits and Risks [Internet]. U.S. Food and Drug Administration. (2017). Available from: https://www.fda.gov/radiation-emitting-products/mri-magnetic-resonance-imaging/benefits-and-risks (accessed November 7, 2021).

[44] Green JJ Berger JS Kramer CM Salerno M. Prognostic value of late gadolinium enhancement in clinical outcomes for hypertrophic cardiomyopathy. JACC Cardiovasc Imaging. (2012) 5:370–7. 10.1016/j.jcmg.2011.11.021 22498326

[45] Österberg AW Östman-Smith I Jablonowski R Carlsson M Green H Gunnarsson C. High ECG risk-scores predict late gadolinium enhancement on magnetic resonance imaging in HCM in the young. Pediatr Cardiol. (2021) 42:492–500. 10.1007/s00246-020-02506-9 33515326

[46] Chen S Wang X Huang L Chen Y Zhang Q. Performance of 12-lead electrocardiogram Selvester QRS scoring criteria to diagnose myocardial scar in patients with hypertrophic cardiomyopathy. Ann Noninvasive Electrocardiol. (2020) 25:e12762. 10.1111/anec.12762 32378804 PMC7507423

[47] Zhang C Liu R Yuan J Cui J Hu F Yang W. Predictive values of N-Terminal Pro-B-Type natriuretic peptide and cardiac troponin I for myocardial fibrosis in hypertrophic obstructive cardiomyopathy. PLoS ONE. (2016) 11:e0146572. 10.1371/journal.pone.0146572 26765106 PMC4713160

[48] Ngo D Sinha S Shen D Kuhn EW Keyes MJ Shi X. Aptamer-based proteomic profiling reveals novel candidate biomarkers and pathways in cardiovascular disease. Circulation. (2016) 134:270–85. 10.1161/CIRCULATIONAHA.116.021803 27444932 PMC4963294

[49] Cheng JM Akkerhuis KM Meilhac O Oemrawsingh RM Garcia-Garcia HM van Geuns R-J. Circulating osteoglycin and NGAL/MMP9 complex concentrations predict 1-year major adverse cardiovascular events after coronary angiography. Arterioscler Thromb Vasc Biol. (2014) 34:1078–84. 10.1161/ATVBAHA.114.303486 24651681

[50] Reiser H Klingenberg R Hof D Cooksley-Decasper S Fuchs N Akhmedov A. Circulating FABP4 is a prognostic biomarker in patients with acute coronary syndrome but not in asymptomatic individuals. Arterioscler Thromb Vasc Biol. (2015) 35:1872–9. 10.1161/ATVBAHA.115.305365 26069234

[51] Zhang Z-Y Thijs L Petit T Gu Y-M Jacobs L Yang W-Y. Urinary proteome and systolic blood pressure as predictors of 5-year cardiovascular and cardiac outcomes in a general population. Hypertension. (2015) 66:52–60. 10.1161/HYPERTENSIONAHA.115.05296 26063667

[52] Berezin AE Kremzer AA Martovitskaya Y V Samura TA Berezina TA Zulli A. The utility of biomarker risk prediction score in patients with chronic heart failure. Int J Clin Exp Med. (2015) 8:18255–64. 10.1186/s40885-016-0041-1 26770427 PMC4694327

[53] Lemesle G Maury F Beseme O Ovart L Amouyel P Lamblin N. Multimarker proteomic profiling for the prediction of cardiovascular mortality in patients with chronic heart failure. PLoS ONE. (2015) 10:e0119265. 10.1371/journal.pone.0119265 25905469 PMC4408082

[54] Hathout Y Brody E Clemens PR Cripe L DeLisle RK Furlong P. Large-scale serum protein biomarker discovery in Duchenne muscular dystrophy. Proc Natl Acad Sci U S A. (2015) 112:7153–8. 10.1073/pnas.1507719112 26039989 PMC4466703

[55] Shi T Moravec CS Perez DM. Novel proteins associated with human dilated cardiomyopathy: selective reduction in α(1A)-adrenergic receptors and increased desensitization proteins. J Recept Signal Transduct Res. (2013) 33:96–106. 10.3109/10799893.2013.764897 23384050 PMC3624731

[56] Frustaci A Ciccosanti F Chimenti C Nardacci R Corazzari M Verardo R. Histological and proteomic profile of diabetic versus non-diabetic dilated cardiomyopathy. Int J Cardiol. (2016) 203:282–9. 10.1016/j.ijcard.2015.10.119 26519687

[57] Sonnenschein K Fiedler J de Gonzalo-Calvo D Xiao K Pfanne A Just A. Blood-based protein profiling identifies serum protein c-KIT as a novel biomarker for hypertrophic cardiomyopathy. Sci Rep. (2021) 11:1755. 10.1038/s41598-020-80868-z 33469076 PMC7815737

[58] Shimada YJ Raita Y Liang LW Maurer MS Hasegawa K Fifer MA. Comprehensive proteomics profiling reveals circulating biomarkers of hypertrophic cardiomyopathy. Circ Heart Fail. (2021) 14:e007849. 10.1161/CIRCHEARTFAILURE.120.007849 34192899 PMC8292216

[59] Larson A Libermann TA Bowditch H Das G Diakos N Huggins GS. Plasma proteomic profiling in hypertrophic cardiomyopathy patients before and after surgical myectomy reveals post-procedural reduction in systemic inflammation. Int J Mol Sci. (2021) 22:2474. 10.3390/ijms22052474 33804404 PMC7957543

[60] Tran DH Wang Z V. Glucose metabolism in cardiac hypertrophy and heart failure. J Am Heart Assoc. (2019) 8:e012673. 10.1161/JAHA.119.012673 31185774 PMC6645632

[61] Kolwicz SCJ Tian R. Glucose metabolism and cardiac hypertrophy. Cardiovasc Res. (2011) 90:194–201. 10.1093/cvr/cvr071 21502371 PMC3078804

[62] Coats CJ Heywood WE Virasami A Ashrafi N Syrris P Dos Remedios C. Proteomic analysis of the myocardium in hypertrophic obstructive cardiomyopathy. Circ Genomic Precis Med. (2018) 11:e001974. 10.1161/CIRCGENETICS.117.001974 30562113

[63] Jain M Brenner DA Cui L Lim CC Wang B Pimentel DR. Glucose-6-phosphate dehydrogenase modulates cytosolic redox status and contractile phenotype in adult cardiomyocytes. Circ Res. (2003) 93:e9–16. 10.1161/01.RES.0000083489.83704.76 12829617

[64] Marsin AS Bertrand L Rider MH Deprez J Beauloye C Vincent MF. Phosphorylation and activation of heart PFK-2 by AMPK has a role in the stimulation of glycolysis during ischaemia. Curr Biol. (2000) 10:1247–55. 10.1016/S0960-9822(00)00742-9 11069105

[65] Depre C Rider MH Veitch K Hue L. Role of fructose 2,6-bisphosphate in the control of heart glycolysis. J Biol Chem. (1993) 268:13274–9. 10.1016/S0021-9258(19)38648-X 8514765

[66] Shimada YJ Batra J Kochav SM Patel P Jung J Maurer MS. Difference in metabolomic response to exercise between patients with and without hypertrophic cardiomyopathy. J Cardiovasc Transl Res. (2020) 14:246–55 10.1007/s12265-020-10051-2 32594362

[67] Aoyagi T Matsui T. Phosphoinositide-3 kinase signaling in cardiac hypertrophy and heart failure. Curr Pharm Des. (2011) 17:1818–24. 10.2174/138161211796390976 21631421 PMC3337715

[68] McMullen JR Shioi T Zhang L Tarnavski O Sherwood MC Kang PM. Phosphoinositide 3-kinase(p110alpha) plays a critical role for the induction of physiological, but not pathological, cardiac hypertrophy. Proc Natl Acad Sci U S A. (2003) 100:12355–60. 10.1073/pnas.1934654100 14507992 PMC218762

[69] DeBosch B Treskov I Lupu TS Weinheimer C Kovacs A Courtois M. Akt1 is required for physiological cardiac growth. Circulation. (2006) 113:2097–104. 10.1161/CIRCULATIONAHA.105.595231 16636172

[70] Bass-Stringer S Tai CMK McMullen JR. IGF1-PI3K-induced physiological cardiac hypertrophy: Implications for new heart failure therapies, biomarkers, and predicting cardiotoxicity. J Sport Heal Sci. (2020) 10:637–47. 10.1016/j.jshs.2020.11.009 33246162 PMC8724616

[71] Hunter JJ Tanaka N Rockman HA Ross JJ Chien KR. Ventricular expression of a MLC-2v-ras fusion gene induces cardiac hypertrophy and selective diastolic dysfunction in transgenic mice. J Biol Chem. (1995) 270:23173–8. 10.1074/jbc.270.39.23173 7559464

[72] Rauen KA Schoyer L McCormick F Lin AE Allanson JE Stevenson DA. Proceedings from the 2009 genetic syndromes of the Ras/MAPK pathway: From bedside to bench and back. Am J Med Genet A. (2010) 152A:4–24. 10.1002/ajmg.a.33183 20014119 PMC4051786

[73] Chen H Li X Liu X Wang J Zhang Z Wu J. Clinical and mutation profile of pediatric patients with RASopathy-associated hypertrophic cardiomyopathy: results from a Chinese cohort. Orphanet J Rare Dis. (2019) 14:29. 10.1186/s13023-019-1010-z 30732632 PMC6367752

[74] Andelfinger G Marquis C Raboisson M-J Théoret Y Waldmüller S Wiegand G. Hypertrophic cardiomyopathy in Noonan syndrome treated by MEK-Inhibition. J Am Coll Cardiol. (2019) 73:2237–9. 10.1016/j.jacc.2019.01.066 31047013 PMC6916648

[75] Wu X Simpson J Hong JH Kim K-H Thavarajah NK Backx PH. MEK-ERK pathway modulation ameliorates disease phenotypes in a mouse model of Noonan syndrome associated with the Raf1(L613V) mutation. J Clin Invest. (2011) 121:1009–25. 10.1172/JCI44929 21339642 PMC3049402

[76] Dhandapany PS Fabris F Tonk R Illaste A Karakikes I Sorourian M. Cyclosporine attenuates cardiomyocyte hypertrophy induced by RAF1 mutants in Noonan and LEOPARD syndromes. J Mol Cell Cardiol. (2011) 51:4–15. 10.1016/j.yjmcc.2011.03.001 21440552 PMC3103595

[77] Marin TM Keith K Davies B Conner DA Guha P Kalaitzidis D. Rapamycin reverses hypertrophic cardiomyopathy in a mouse model of LEOPARD syndrome-associated PTPN11 mutation. J Clin Invest. (2011) 121:1026–43. 10.1172/JCI44972 21339643 PMC3049377

[78] Mayer IA Arteaga CL. The PI3K/AKT pathway as a target for cancer treatment. Annu Rev Med. (2016) 67:11–28. 10.1146/annurev-med-062913-051343 26473415

[79] Song HK Kim J Lee JS Nho KJ Jeong HC Kim J. Pik3ip1 modulates cardiac hypertrophy by inhibiting PI3K pathway. PLoS ONE. (2015) 10:e0122251. 10.1371/journal.pone.0122251 25826393 PMC4380398

[80] Captur G Heywood WE Coats C Rosmini S Patel V Lopes LR. Identification of a multiplex biomarker panel for hypertrophic cardiomyopathy using quantitative proteomics and machine learning. Mol Cell Proteomics. (2020) 19:114–127. 10.1074/mcp.RA119.001586 31243064 PMC6944230

